# Pretest Score for Predicting Microbubble Contrast Agent Use in Stress Echocardiography: A Method to Increase Efficiency in the Echo Laboratory

**DOI:** 10.4061/2009/308486

**Published:** 2009-07-21

**Authors:** Mathieu Bernier, Sahar S. Abdelmoneim, Stuart Moir, Robert B. McCully, Patricia A. Pellikka, Sharon L. Mulvagh

**Affiliations:** ^1^Cardiovascular Ultrasound Imaging Laboratory, Laval Hospital, QC, Canada G1V 4G5; ^2^Division of Cardiovascular Diseases, Mayo Clinic, Rochester, Minnesota 55905, USA

## Abstract

*Background*. In stress echocardiography, contrast agents are used selectively to improve endocardial border definition. Early identification of candidates may facilitate use of these agents in small and medium volume laboratories where resources are limited. *Methods*. We studied 15232 patients who underwent stress echocardiography. Contrast agent was used if 2 or more ventricular segments were not adequately visualized without contrast. Logistic regression models were used to evaluate the association between individual characteristics and contrast use. An 11-point score was derived from the significant characteristics. *Results*. Variables associated with microbubble use were age, sex, smoking, presence of multiple risk factors, bodymass index (BMI), referral for dobutamine stress echocardiography, history of coronary artery disease, and abnormal baseline electrocardiogram. All variables except BMI were given a score of 1 if present and 0 if absent; BMI was given a score of 0 to 4 according to its value. An increased score was directly proportional to increased likelihood of contrast use. The score cutoff value to optimize sensitivity and specificity was 5. *Conclusions*. A pretest score can be computed from information available before imaging. It may facilitate contrast agent use through early identification of patients who are likely to benefit from improved endocardial border definition.

## 1. Introduction

 Obtaining a satisfactory image in patients with certain characteristics, including obesity, chest wall deformities, and severe obstructive pulmonary disease, continues to be a challenge in diagnostic echocardiography. In these patients, stress echocardiography presents an even greater challenge because definition of the endocardial border is often worse during or immediately after the applied stress [1]. This reduced endocardial border definition is partly due to increased chest wall motion related to tachypnea and heart movement due to tachycardia. With fundamental imaging, as many as 30% of studies show inadequate endocardial definition [2] and may be nondiagnostic.

**Figure 1:**
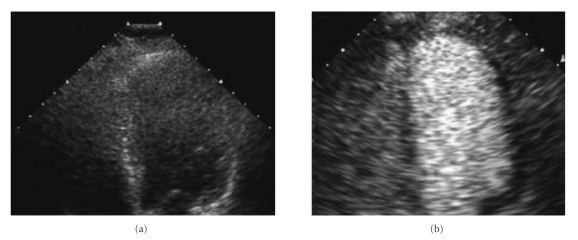
(a) Apical 4-chamber view (left ventricle on right, end-diastole) with harmonic imaging. (b) Same view with enhancement due to intravenous microbubble contrast agent. Image shows marked improvement of endocardial border definition, especially at apex and in mid segment of anterolateral wall.

Numerous transpulmonary microbubble contrast agents have been shown to have an incremental benefit over harmonic imaging for detection of endocardial border in selected patients and have now been approved by the US Food and Drug Administration for left ventricular opacification [1, 3]. Use of microbubble contrast agents in stress echocardiography improves endocardial border definition and accuracy to a degree in which a technically “inadequate” study with incomplete endocardial visualization can be rendered interpretable and have sensitivity and specificity comparable to an “adequate” study [4]. Figure 1 shows an example of inadequate endocardial border definition with harmonic imaging improved with microbubble contrast agent use.

Candidates for use of microbubble contrast agents during stress echocardiography are usually identified on the basis of suboptimal or inadequate resting images. Performance of a contrast echocardiographic study requires that (1) approval for contrast use is obtained from a physician, (2) intravenous access is achieved, and (3) the presence of appropriately qualified personnel are available. This sequence of events may increase procedure time, decrease laboratory throughput, and reduce patient satisfaction. Thus, some centers have adopted strategies to decrease the time required to initiate a contrast study. An example of such a strategy is the development of a standing order that allows a qualified nurse or sonographer to proceed directly with the preparation and administration of microbubble contrast agents for left ventricular opacification [5, 6]. However, such a strategy relies on the initial acquisition of resting images and consumes time, termed *struggle time*, before a decision is made to proceed [7]. The use of intravenous microbubble contrast agents has previously been shown to be accurate and cost-effective [4, 8]. We sought to test predictors of contrast agent use and to develop a simple score that integrates those predictors. We required that the score was easily calculated at patient presentation and was readily computable before any image acquisition.

## 2. Methods

### 2.1. Population

We identified 16 052 consecutive patients who had a clinically indicated stress echocardiogram at our laboratory between November 2003 and November 2005. Information on the use of microbubble contrast agents was available in 15 253 patients (95%); the data from these patients were included in the analysis. From November 2003 to July 2004, the decision to use a microbubble contrast agent was made on an individual, case-by-case basis whereby the physician reviewed resting images and determined need. From July 2004, a guideline policy was implemented, enabling the imaging sonographer to determine the need for microbubble use on the basis of observed resting image quality. Under this standing order, a microbubble contrast agent was used if reasonable attempts had been made to optimize the echocardiographic images and the endocardium of 2 or more left ventricular segments was not visualized adequately to evaluate cardiac structure in systole and diastole. This decision, in turn, triggered implementation of a protocol for immediate intravenous access and preparation of a contrast agent, initiated by a page to a central number to notify the assigned individual from a team of echocardiography nurses. The nurse then prepared the contrast agent, obtained intravenous access, and, when requested by the sonographer, administered the contrast agent. (All registered nurses and sonographers working in the stress echocardiography area had been trained in contrast agent administration and in optimization of image acquisition according to online, real-time image appearance.) From July 2004 to November 2005, the contrast agents perflutren protein-type A microspheres (Optison; Amersham Health, Inc, Princeton, New Jersey) and perflutren lipid microspheres (Definity; Bristol-Myers Squibb Medical Imaging Inc, North Billerica, Massachusetts) were used on alternate months, to enable the standing order. Before this time, contrast agent selection was at the discretion of the ordering physician.

### 2.2. Measurements

All clinical data were prospectively entered into a database at the time of the stress echocardiogram. Demographic variables such as sex and age were collected. Information on a self-reported history of coronary artery disease (CAD) (e.g., prior myocardial infarction, percutaneous, or surgical revascularization) was obtained. Weight and height were measured and body mass index (BMI) was calculated. BMI was unavailable for 202 patients (1%). A resting electrocardiogram was considered abnormal if it contained pathologic Q waves, resting ST-T wave abnormalities, left bundle branch block, or left ventricular hypertrophy. The stress-testing methods included echocardiography with dobutamine or treadmill exercise. Major risk factors for CAD were also identified, including hypertension, diabetes, a family history of CAD, and hyperlipidemia. Smoking status was evaluated but considered separately. Since smoking is associated with chronic obstructive pulmonary disease and, hence, with poor quality images, we postulated that this variable could be used to predict microbubble contrast agent use. Use of *β*-blockers and calcium channel blockers was also recorded. Table 1 shows clinical characteristics of the cohort. 

### 2.3. Statistical Analysis

Continuous variables are presented as mean ± SD; discrete variables are presented as frequency distribution. Means were compared using 2-sample independent *t* test, and categorical variables were compared using conventional *χ* ^2^ testing. A multivariate logistic regression model was built to evaluate the statistical significance of the association between identified pretest variables and use of a microbubble contrast agent during stress echocardiography. Variables were tested individually in a univariate model, and those that achieved a level of significance (*P* < .20) were tested in a multivariate stepwise logistic regression.

BMI was represented by 4 categorical variables; hence, no values for univariate analysis were reported. However, univariate analysis is included for BMI measured on a continuous scale. Variables with significance (*P* < .05) in multivariate analysis were kept in the final score model. SAS PROC LOGISTIC (SAS Institute Inc, Cary, North Carolina) was used to obtain maximum likelihood estimates of the parameter coefficients. Multiplicative interaction between sex and BMI and smoking and BMI, as well as between type of stress and BMI, was tested and did not show statistical significance. Accordingly, no interaction terms were retained in the final model. The model's goodness of fit was assessed using the Hosmer-Lemeshow test. A low *χ* ^2^ statistic with a high *P* value indicates that the logistic function fits the data adequately.

For our score, we classified BMI (kg/m^2^) into 5 categories with associated point allocation: BMI <25, 0 point; BMI 25 to 30, 1 point; BMI >30 to 35, 2 points; BMI >35 to 40, 3 points; and BMI >40, 4 points. Electrocardiogram anomalies, smoking, referral for dobutamine stress echocardiography, major cardiovascular risk factors (more than 2 and other than smoking), a previous history of CAD, and age 65 years and older were transformed to binary variables (i.e., 1 if present and 0 if not). An arbitrary cutoff point of 65 years was chosen for age. This cutoff was close to the cohort's mean age, and changing the cutoff point by adding or subtracting 10 years did not change the odds ratio appreciably.

The derived score was simply the arithmetic sum of the predictive variables of the model, and an increasing weight was accorded to an increasing BMI. Scores ranged from 0 to 11. The increasing cumulative incidence proportion of contrast agent use according to the score was tested with *χ* ^2^ for trend (Cochran-Armitage). A univariate logistic regression of the total score as a predictor of microbubble contrast agent use was also performed. Internal validation was conducted by bootstrap repeated-sampling method (1000 samples with replacement), and predictors of coefficients from the samples were recorded. Mean and standard error of these coefficients were compared with those obtained from the full cohort to confirm the robustness of the model. 

## 3. Results

**Figure 2:**
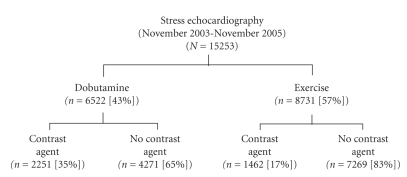
Flowchart of patients included in analyses.

The primary outcome, defined as the use of a microbubble contrast agent during stress echocardiography, occurred in 3713 (24%) of the 15 253 patients in the study group. A microbubble contrast agent was used in 2251 patients (35%) of the group undergoing dobutamine stress echocardiography and in 1467 (17%) of the group undergoing exercise stress echocardiography (*P* < .001) (Figure 2). The cumulative incidence proportion of contrast agent use significantly differed according to the period for which it was calculated. From November 2003 to July 2004, when the decision to use a contrast agent depended on physician review of resting images and direct order to give a contrast agent, the cumulative incidence proportion was 15.6%, compared with 28.5% from July 2004 to November 2005, when the decision to give a contrast agent was sonographer-driven, based on standing protocol (*P* < .001). Image quality was judged to be improved by the use of contrast in 3445 cases (93%), unchanged in 215 cases (6%), and worsened in 53 cases (1%). Thirteen predictors were tested in the multivariate analysis and 11 variables were retained in the final score model, including 4 categorical variables for BMI (Table 2). The Hosmer-Lemeshow statistic was 10.89 (*P* = .21), reflecting an appropriate fit of the model. The allocated score for every variable finally retained is shown in Table 2. Because of increased strength of association with higher BMI and outcome, an increasing score weight was allocated to increasingly higher BMI levels. Regression coefficients and odds ratios for BMI as continuous variables as well as other variables that were not retained in the final model are presented in Table 3.

**Figure 3:**
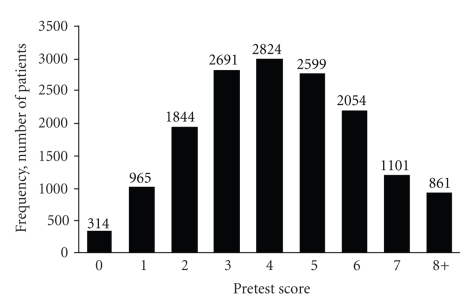
Distribution of pretest scores in the cohort of 15,253 patients. “8+” indicates an individual score of 8, 9, 10, or 11.

**Figure 4:**
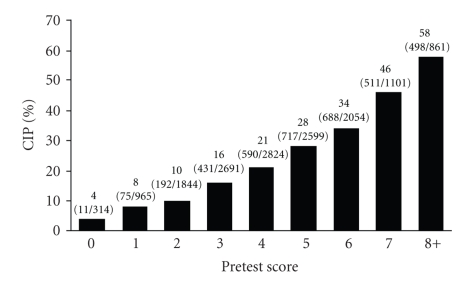
Cumulative incidence proportion (CIP) of contrast agent use according to pretest score. Below the *x*-axis label, the denominators indicate the number of patients with each score and the numerators indicate the corresponding subset of patients who had contrast agent use. “8+” indicates an individual score of 8, 9, 10, or 11.

**Figure 5:**
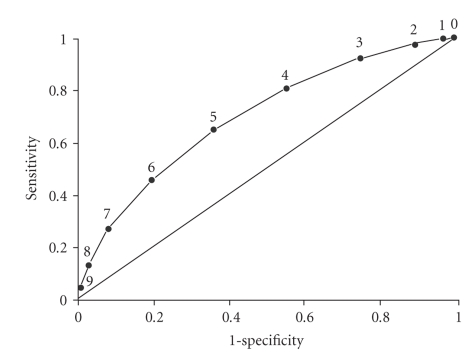
Receiver operating characteristic curve for total pretest score. Area under the curve is 0.70.

All significant variables showed a positive association with microbubble contrast agent use. BMI (particularly BMI >40) and dobutamine stress echocardiography were the 2 variables most likely to predict outcome. A clear gradient of association existed between increasing BMI and outcome. In addition, the computed score in univariate analysis was found to be a good predictor of outcome. Figure 3 shows the distribution of the scores, which followed a normal distribution pattern; Figure 4 shows the relation between computed score and cumulative incidence proportion of contrast agent use (Cochran-Armitage test for trend, *P* < .001). Because the number of patients who had an individual score of 8, 9, 10, or 11 was small, these patients were combined into 1 group labeled “8+.” The cumulative incidence proportion for use of contrast agent was 8% for a score of 1, 21% for 4, and 58% for 8+ (*P* for trend, <.001). Receiver operator characteristic curves analysis was performed (Figure 5). A cutoff score of 5 had the best discriminative properties—66% sensitivity and 64% specificity—to correctly identify individuals who required intravenous contrast agents. Area under the curve was 0.70. 

## 4. Discussion

 Our results suggest that clinical information, which is routinely available at presentation for the stress echocardiography procedure, can be used to identify patients in whom the use of intravenous microbubble contrast agents is likely to be needed. This information, including age, sex, smoking, presence of risk factors for CAD, history of CAD, electrocardiogram anomalies, and referral for dobutamine stress echocardiography can easily be determined before imaging. Admittedly, our observation of frequent use in obese patients was not that surprising. Obese patients are often difficult to image, and the finding of a positive association between a high BMI and the use of microbubble contrast agents is consistent with intuitive knowledge. Similarly, the more frequent use of microbubble contrast agents during dobutamine stress echocardiography might be explained by the preordained availability of requisite intravenous access and trained personnel. However, during treadmill echocardiographic stress testing, poststress image acquisition is frequently more challenging than during dobutamine echocardiographic stress testing because of limited image acquisition time and frequent hyperventilation. Unfortunately, the quality of the baseline resting images cannot always predict the quality of the postexercise images. Therefore, awareness of need for microbubble contrast agent use at presentation for exercise stress testing would be very useful.

The positive association found between an abnormal baseline electrocardiogram, the presence of multiple risk factors, and microbubble contrast agent use is interesting. These variables are not likely to be causally associated with poor endocardial border definition per se, but a plausible explanation is that their presence is associated with a higher prevalence of CAD and, hence, wall motion abnormalities. To increase the level of confidence in interpretation of wall motion in the presence of either an abnormal baseline electrocardiogram or multiple risk factors, echocardiography consultants may be more likely to use a microbubble contrast agent.

The approach used to devise our prediction score system is similar to that of Croft et al. [9], who developed a tool to clinically predict nerve function impairment in leprosy patients, and is also derived from Antman et al. [10], who published the Thrombosis in Myocardial Infarction risk score for unstable angina and non-ST-elevation myocardial infarction.

Our contrast use prediction score was designed to be easily computable before imaging and simple to remember. In low- or medium-volume centers where contrast implementation is yet evolving, this screening method could allow more efficient resource distribution and could be used to guide procedural flow at different levels of intensity. For example, the availability of appropriately trained personnel to perform contrast imaging studies could be facilitated by designating an area where patients preidentified with screening could be preferentially directed, and if 2-dimensional imaging was confirmed to be inadequate, contrast agents could be used without further delay. Ideally, the score would be used to identify candidate patients, and intravenous access would be placed even before imaging was initiated. This strategy could potentially be most useful in patients who are scheduled to have exercise stress testing, because the quality of resting images may not predict that of the postexercise images. 

Our results are derived from a high volume stress echocardiography laboratory with sonographers accustomed to take the decision to directly proceed to contrast usage or not. Clinical practice patterns, sonographers familiarity with contrast usage, and even patient population may vary significantly from center to center and could possibly limit the applicability of our method in certain circumstances. 

 Furthermore, given the discriminatory potential of our score, a proportion of patients would have a misclassified prediction of microbubble contrast agent use. To overcome this limitation, a higher cutoff point could be used to make a decision. A cutoff score of 7 or more would lead to a specificity of 91% at the price of a sensitivity of 27%. With the objective of the score being to quickly identify individuals most likely to require microbubble contrast agent use, specificity could be favored because contrast agents can be given later and as needed on the basis of the results of the initial imaging. This strategy would be most relevant in patients presenting for dobutamine stress testing, in whom intravenous access is already present and only the decision to administer a contrast agent needs to be made. The implications of inappropriately directing a patient to a room where all equipment settings are ready for contrast imaging are negligible. If, on the basis of resting images, it is determined that the patient does not need a microbubble contrast agent, clinical procedures will not be delayed, no increase in cost will be incurred, and the patient will not be exposed to any additional risk. Implementation of the contrast use prediction score should allow gains in efficiency and facilitate the appropriate use of contrast agents when indicated. 

## Figures and Tables

**Table 1:** Clinical characteristics of the studied population of patients.

Characteristic	Value* (*N* = 15, 253)
Age, *y*	64.4 ± 13.4
Male sex	8,251 (54)
Body mass index	28.5 ± 6.1
Dobutamine stress echocardiography	6,522 (43)
Resting ejection fraction, %	59 ± 8
Smoking (current or past)	7,862 (52)
Major risk factors (>2, smoking excluded)	4,282 (28)
Diabetes	2,811 (18)
Family history of CAD	5,853 (38)
Hypertension	9,022 (59)
Dyslipidemia	9,655 (63)
Known CAD (e.g., angina, MI, revascularization)	3,372 (22)
Prior CABG	1,455 (10)
Prior PCI	1,754 (11)
Prior MI	1,809 (12)
Abnormal ECG	7,663 (50)
Left bundle branch block	406 (3)
Pathologic Q waves	1,333 (9)
Resting ST-T wave abnormalities	7,257 (48)
Left ventricular hypertrophy	581 (4)
*β*-Blocker use	6,030 (40)
Calcium channel blocker use	2,665 (17)
Contrast agent use	3,713 (24)

CABG, coronary artery bypass graft; CAD, coronary artery disease; ECG, electrocardiogram; MI, myocardial infarction; PCI, percutaneous coronary intervention.

*Categorical data are expressed as number of patients and percentage of sample; continuous data are expressed as mean ± SD.

**Table 2:** Significant predictors of contrast agent use included in the final score model and the allocated score values.

Predictor	Univariate analysis			Multivariate analysis			Allocated score
	*β* Coefficient	*P* value	OR (95% CI)	*β* Coefficient	*P* value	OR (95% CI)	
Age ≥65 *y*	0.38	<.001	1.46 (1.35–1.57)	0.28	<.001	1.32 (1.21–1.44)	1
Male sex	0.21	<.001	1.23 (1.14–1.32)	0.21	<.001	1.23 (1.13–1.34)	1
Smoking	0.25	<.001	1.29 (1.2–1.39)	0.12	.003	1.13 (1.04–1.23)	1
Multiple (>2) risk factors for CAD*	0.5	<.001	1.65 (1.53–1.79)	0.13	.005	1.13 (1.04–1.24)	1
BMI^†‡^							
<25							0
25–30	—	—	—	0.57	<.001	1.76 (1.57–1.98)	1
>30–35	—	—	—	1.33	<.001	3.79 (3.35–.26)	2
>35–40	—	—	—	1.78	<.001	5.94 (5.11–6.90)	3
>40	—	—	—	2.12	<.001	8.36 (6.92–10.09)	4
Dobutamine stress echocardiography^§^	0.96	<.001	2.62 (2.43–2.83)	0.76	<.001	2.13 (1.96–2.32)	1
Previous history of CAD^//^	0.49	<.001	1.63 (1.50–1.78)	0.31	<.001	1.36 (1.23–1.50)	1
Abnormal ECG^¶^	0.28	<.001	1.32 (1.23–1.43)	0.11	.01	1.11 (1.03–1.21)	1

BMI, body mass index; CAD, coronary artery disease; CI, confidence interval; ECG, electrocardiogram; OR, odds ratio.

*Risk factors include hypertension, diabetes, hyperlipidemia, and family history of CAD.

^†^Ellipses indicate not applicable.

^‡^Reference category is a BMI lower than 25.

^§^Results of dobutamine stress echocardiography compared with results of treadmill stress echocardiography.

^//^Includes prior myocardial infarction and prior percutaneous coronary intervention or coronary artery bypass graft.

^¶^Includes those showing left ventricular hypertrophy, pathologic Q waves, left bundle branch block, or resting ST-T wave abnormalities.

**Table 3:** Tested variables not included in the final score model.

	Univariate analysis			Multivariate analysis		
	*β* Coefficient	*P* value	OR (95% CI)	*β* Coefficient	*P* value	OR (95% CI)
Calcium channel blocker use	0.18	.003	1.19 (1.08–1.32)	−0.06	.33	0.95 (0.85–1.05)
*β*-Blocker use	0.41	<.001	1.5 (1.39–1.62)	0.07	.11	1.07 (0.98–1.17)
BMI (per 10-unit increase)	1.06	<.001	2.88 (2.67–3.08)	1.04	<.001	2.83 (2.64–3.04)
Total score* (per 2-unit increase)	0.84	<.001	2.31 (2.20–2.42)	—	—	—

BMI, body mass index; CI, confidence interval; OR, odds ratio.

*Ellipses indicate not applicable.
